# Supplementary material for: Imaging magnetic transition of magnetite to megabar pressures using quantum sensors in diamond anvil cell
Source: Nat Commun. 2024 Oct 14;15:8843. doi: 10.1038/s41467-024-52272-y (PMC11471789; doi:10.1038/s41467-024-52272-y)
Supplement: Supplementary file 1 — Supplementary Information [file 41467_2024_52272_MOESM1_ESM.pdf]

Supplementary Information

Authors:

Mengqi Wang[1,2,3]†, Yu Wang[4,5]†*, Zhixian Liu[1,2,3]†, Ganyu Xu[1,2,3], Bo Yang[1,2,3], Pei Yu[1,2,3], Haoyu Sun[1,2,3], Xiangyu Ye[1,2,3], Jingwei Zhou[1,2,3,6], Alexander. F. Goncharov[7], Ya Wang[1,2,3,6]* and Jiangfeng Du[1,2,3,6,8]*

Affiliations:

[1] CAS Key Laboratory of Microscale Magnetic Resonance and School of Physical Sciences, University of Science and Technology of China, Hefei 230026, China

[2] Anhui Province Key Laboratory of Scientific Instrument Development and Application, University of Science and Technology of China, Hefei 230026, China

[3] CAS Center for Excellence in Quantum Information and Quantum Physics, University of Science and Technology of China, Hefei 230026, China

[4] Key Laboratory of Materials Physics, Institute of Solid State Physics, HFIPS, Chinese Academy of Sciences, Hefei, China.

[5] Institute of Geosciences, Goethe University Frankfurt, Frankfurt 60438, Germany

[6] Hefei National Laboratory, University of Science and Technology of China, Hefei 230088, China

[7] Earth and Planets Laboratory, Carnegie Institution of Washington, Washington, DC, USA.

[8] Institute of Quantum Sensing and School of Physics, Zhejiang University, Hangzhou 310027, China

*Corresponding author. Email: wangyu@issp.ac.cn (Yu Wang), ywustc@ustc.edu.cn (Ya Wang) and djf@ustc.edu.cn (Jiangfeng Du)

†These authors contributed equally to this work

CONTENTS

1 Supplementary Methods: 3
  1.1 Experiment setup 3
  1.2 DAC preparing and sample loading process 4
  1.3 Pressure calibration and magnetic field imaging process 5
2 Supplementary Table 1: Sensitivity and pressure range 6
3 Supplementary Note 1: Stress tensor 8
  3.1 Hamiltonian in NV frame 8
  3.2 Stress tensor of the uniaxial stress ($\boldsymbol{\sigma}$) 9
  3.3 Stress susceptibility parameters 10
  3.4 Shift of the zero-field splitting Ds with stress. 11
  3.5 Peak intensity asymmetry in ODMR 12
4 Supplementary Note 2: Impact of uniaxial stress on [111] and non-[111] NV centers. 15
5 Supplementary Note 3: Experiments with diamond particles loaded inside a high-pressure chamber 18
6 Supplementary Note 4: Magnetic signals and error analysis 20
  6.1 Extracting the magnetic field B_z^{mag} induced by magnetite from the Reference NV. 20
  6.2 Uniformity of the external magnetic field 23
  6.3 Relative error for weak magnetic field approximation $(\Delta\mathrm{sim} - \Delta)/\Delta$ 23
  6.4 Relative deviation for $\Delta_B \gg \Delta_{\sigma_\perp}$ condition 25
  6.5 Analysis and discuss for ODMR spectra linewidth broadening $\Gamma\mathrm{mag}$ 27
References in Supplementary 30

1 Supplementary Methods:

1.1 Experiment setup

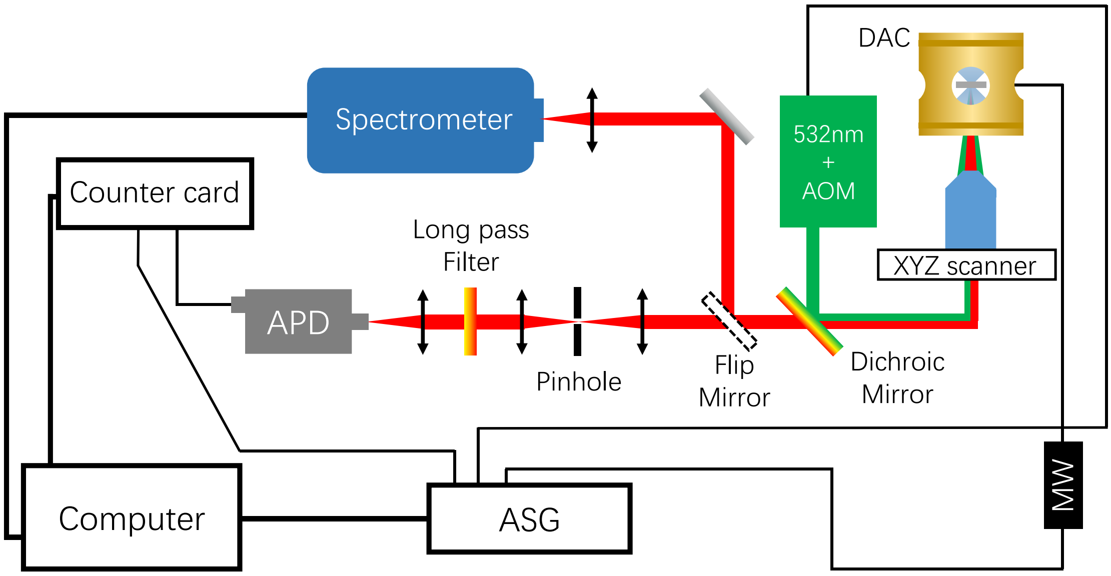

Supplementary Fig. 1. Schematic drawing of the ODMR setup. MW: Microwave source; AOM: Acousto-optic modulator; APD: Avalanche photodiode single-photon detector; ASG: Arbitrary sequence generator; DAC: Diamond anvil cell

A home-built ODMR setup was used to address NV ensembles inside the DAC and collect fluorescence signals under extreme pressure (Supplementary Fig. 1). For initializing NV ensembles, a 532 nm laser (CNILaser MGL-III-532-150mW) was controlled by two serial acousto-optic modulators (AOM Gooch&Housego AOMO 3200-121). The laser beam was focused on the anvil tip through the optical window of the DAC using a long working distance objective lens (Mitutoyo MY20X-824, NA0.4), which was mounted on a piezo scanning stage (Coremorrow P12A.XYZ100S). Through the same objective lens, the NV fluorescence was separated from the excitation laser beam using a dichroic mirror. A flip mirror was used to switch the light path between a spectrometer (Horiba iHR550) for pressure measurement and the confocal system, which consisted of a 50 μm pinhole and a 633 nm long-pass filter (Semrock BSP01-633R-25). The fluorescence signal was detected using a single photon counting module (Excelitas SPCM-780-24-BR1), and a counter card (National

Instruments PCIe-6612) was used for photon signal counting. The optical diffraction limit is given by the formula: $r = 0.61\,\lambda/NA$, where $\lambda = 523$ nm is the wavelength of the excitation light, and $NA = 0.4$ is the numerical aperture of the objective lens used. And the spatial resolution r is evaluated to be approximately 0.8 μm. For NV spin manipulation, a microwave (MW) source (Rigol DSG3060) combined with a 50W amplifier (Mini-Circuits ZHL-50W-63+) was employed to generate the MW signal. The entire system was controlled by a Pulse Generator (CIQTEK ASG8100), which provided pulse sequences for timing control.

1.2 DAC preparing and sample loading process

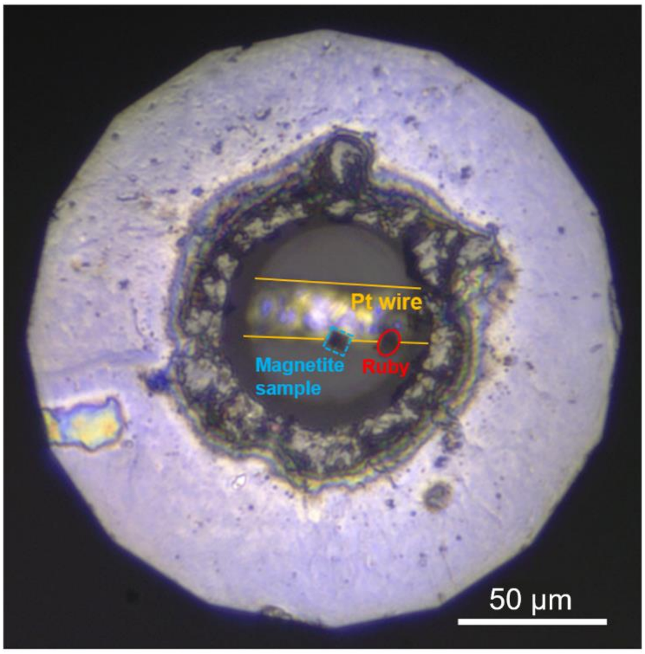

Supplementary Fig. 2. Photographs of sample distribution after sample loading process

In our experiments, diamond anvils with [111]-crystal cut culets were used. Both 100 μm and 150 μm diameter culets were used in this work. A 250 μm thick rhenium (Re) gasket was indented to about 20-25 GPa using a nonmagnetic BeCu symmetric DAC equipped with standard-design BeCu seats. The bottom of the imprint was drilled out, and a mixture of nano cBN powder with epoxy was placed in the imprint. It was then pressed between the anvils to form an insulating layer that isolated the platinum (Pt) foil from the Re gasket. A hole with a diameter of 30-60 μm was drilled at the

center of the insulating layer. Using a micromanipulator (MicroSupport QP-3RH), the magnetite sample and ruby particles were placed inside the sample chamber on the surface of the culet with NV centers. The sample chamber was then filled with KCl, a pressure-transmitting medium (PTM), to provide the quasi-hydrostatic environment. A 10 μm thick platinum (Pt) wire was placed across the sample chamber to apply microwave radiation to control the spin states. The ends of the Pt wire were led out with copper wires, and the joints were glued with conductive silver paste.

1.3 Pressure calibration and magnetic field imaging process

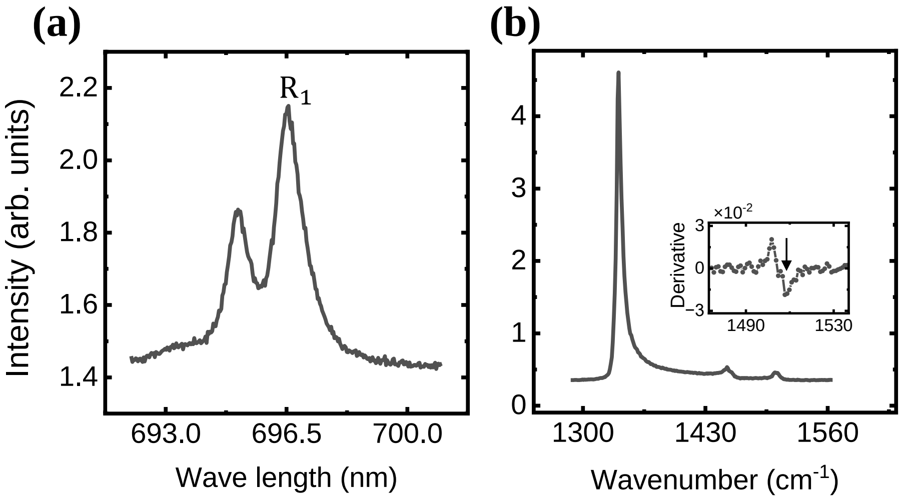

Supplementary Fig. 3. The Ruby fluorescence and the diamond culet Raman spectra for pressure calibration. (a) fluorescence of the ruby inside the sample chamber with pressure of 6.6 GPa. (b) Raman spectrum of the culet of the diamond anvil with pressure of 85.1 GPa, the inset shows the derivative of the high-frequency edge of the Raman band and marked by an arrow.

The shift of the ruby R_1 luminescent line (below 20 GPa)[1] and the high-frequency edge of the Raman band of the diamond (above 20 GPa)[2] were used as pressure calibrants. It's important to consider the presence of stress gradients in the pressure chamber and the impact of uniaxial stress on the culet of the (111)-cut diamond anvil, which may introduce an error of a few Giga pascal in the pressure calibration.[3]

To perform magnetic field imaging, a grid with dimensions of 14×10 and a spacing of 500 nm between each grid pixel was established. For each pixel, an ODMR was conducted to obtain the local magnetic field by fitting the full ODMR spectral. The

accurate positioning was realized through the piezo scanning stage (Coremorrow P12A.XYZ100S). Throughout the magnetic imaging experiments, an external magnetic field B^{ext} was maintained to eliminate the influence of stresses. The magnetic field induced by magnetite was extracted by comparing the obtained signal to a reference signal without magnetite. For further information regarding the magnetic signals and error analysis, please refer to Section 6.

2 Supplementary Table 1: Sensitivity and pressure range

In this section, we compare various magnetometry methods based on quantum sensors, as presented in Fig. 2g of the main text. For each method, we estimate the magnetic field sensitivity and plot it against the corresponding pressure. Except as otherwise expressly stated, we obtain the FL contrast and linewidth from the original data source to gather information for our analysis. The shot-noise-limited magnetic field sensitivity of an NV^- magnetometer is estimated by:

$$\eta_B = \frac{4}{3\sqrt{3}}\frac{h}{g_{\text{e}}\mu_{\text{B}}}\frac{\Gamma}{C\sqrt{R}} \tag{1}$$

with Planck constant h, g-factor g_{e}, Bohr magneton μ_{B}, photon-detection rate R, linewidth Γ, and FL contrast C. The pre-factor $4/(3\sqrt{3})$ originates from the Lorentz line shape. Since the photon count rates reported by most of these works are among 1 M~3 Mcts/s, we uniformly set the count rate as 2 Mcts/s, which will have little and tolerable effect on the assessment result. The sensitivity estimation for the work shown in Fig. 2g is shown in Table. S1

Reference	Strategies	Pressure (GPa)	FL contrast (arb. units)	Linewidth (MHz)	Sensitivity ($\mu T/\sqrt{Hz}$)
**Hsieh, et al.** [4]	NV inside anvil	22	1.80%	10	10.80
**Lesik, et al.** [5]	NV inside anvil	7	2.80%	15	10.41
**Yip, et al.** [6]	NV inside chamber	4	2.50%	14	10.89
**Shang, et al.** [7]	NV inside chamber	43	3%	35	27.22
**Doherty, et al.** [8]	NV inside chamber	60.4	0.60%	40	129.60
**Dai, et al.** [9]	NV inside chamber	137.7	0.40%	250	1215.02
**Hilberer, et al.** [10]	Microstructure on anvil	131	1.50%	130	168.48
**Wang, et al.** [11]	SiC_V_{si} on anvil	24.6	0.15%	20	259.21
**Liu, et al.** [12]	SiC_PL_5 on anvil	42	0.01%	15	2916.06
**This work**	NV inside (111)-cut anvil	128.5	30%	20	1.30

Supplementary Table 1. Sensitivity estimation for high-pressure magnetometry techniques based on the color center

3 Supplementary Note 1: Stress tensor

3.1 Hamiltonian in NV frame

In the NV frame ($\hat{x} \parallel [\bar{1}\bar{1}2], \hat{y} \parallel [1\bar{1}0], \hat{z} \parallel [111]$), the Hamiltonian is expressed as follows:

$$H = H_D + H_B + H_S \tag{2}$$

Where

$H_D = D_0 {S_z}^2$ is the zero-field term with a zero-field splitting D_0 = 2.87 GHz.

$H_B = \gamma_e \vec{\mathbf{B}} \cdot \vec{\mathbf{S}}$ is the Zeeman term, where γ_e is the gyromagnetic ratio of the NV center, and $\vec{\mathbf{B}} = (B_x, B_y, B_z)$ is the magnetic field vector.

H_S is a stress evolution term that describes the stress interaction and is given by[13]:

$$H_S = \Pi_z {S_z}^2 + \Pi_x ({S_y}^2 - {S_x}^2) + \Pi_y (S_x S_y + S_y S_x) \tag{3}$$

where $\mathbf{\Pi} = (\Pi_x, \Pi_y, \Pi_z)$ represents the effective electric field associated with mechanical stress and is defined as:

$$\Pi_x = \alpha_2(\sigma_{yy} - \sigma_{xx}) + 2\beta_2\sigma_{xz} \tag{4}$$

$$\Pi_y = 2(\alpha_2\sigma_{xy} + \beta_2\sigma_{yz}) \tag{5}$$

$$\Pi_z = \alpha_1(\sigma_{xx} + \sigma_{yy}) + \beta_1\sigma_{zz} \tag{6}$$

The stress susceptibility parameters, $\alpha_1, \beta_1, \alpha_2,$ and β_2, are defined in the NV frame (See section 3.3)

$D_S = \Pi_z$ is the energy shift of the zero-field splitting produced by the stress comprising hydrostatic pressure (ρ) and uniaxial stress $\sigma_\parallel$ perpendicular to the NV center axis.

$\Delta_{\sigma_\perp} = \sqrt{{\Pi_x}^2 + {\Pi_y}^2}$ is half of the splitting in the $m_s = \pm 1$ spin sublevels produced by the stress component $\sigma_\perp$ (including $\{(\sigma_{yy} - \sigma_{xx}), \sigma_{xz}, \sigma_{xy}, \sigma_{yz}\}$), which breaks the C_{3V} symmetry.

3.2 Stress tensor of the uniaxial stress (σ)

The stress at the anvil tip comprises hydrostatic pressure (ρ) and uniaxial stress (σ) perpendicular to the surface[14]. The hydrostatic pressure (ρ) can be expressed as:

$$\rho = \begin{bmatrix} l & 0 & 0 \\ 0 & l & 0 \\ 0 & 0 & l \end{bmatrix} \tag{7}$$

And the uniaxial stress (σ) in the lab frame ($\hat{X}^{\mathrm{lab}} \parallel [\bar{1}\bar{1}2]$, $\hat{Y}^{\mathrm{lab}} \parallel [1\bar{1}0]$, $\hat{Z}^{\mathrm{lab}} \parallel [111]$) is shown as:

$$\sigma^{(\text{Lab frame})} = \begin{bmatrix} 0 & 0 & 0 \\ 0 & 0 & 0 \\ 0 & 0 & m \end{bmatrix} \tag{8}$$

For NV centers along [111] directions, the NV frame ($\hat{z} \parallel [111]$, $\hat{x} \parallel [\bar{1}\bar{1}2]$, $\hat{y} \parallel [1\bar{1}0]$) coincides with the lab frame. The uniaxial stress (σ) in the NV frame is shown as:

$$\sigma^{(\text{NV [111] frame})} = \overbrace{\begin{bmatrix} 0 & 0 & 0 \\ 0 & 0 & 0 \\ 0 & 0 & m \end{bmatrix}}^{\sigma_{\parallel}} + \overbrace{0}^{\sigma_{\perp}} \tag{9}$$

For NV centers along $[1\bar{1}\bar{1}]$, $[\bar{1}1\bar{1}]$, and $[\bar{1}\bar{1}1]$ directions (not perpendicular to the surface in the (111)-cut anvil), the uniaxial stress (σ) in the NV frame (take NV in $[\bar{1}\bar{1}1]$ directions as an example) is shown as:

$$\sigma^{(\text{NV }[\bar{1}\bar{1}1]\text{frame})} = U\sigma^{(\text{Lab frame})}U^{\mathrm{T}} \tag{10}$$

Where U is the coordinate transformation matrix from Lab frame to NV frame ($\hat{z} \parallel [\bar{1}\bar{1}1]$, $\hat{x} \parallel [112]$, $\hat{y} \parallel [\bar{1}10]$):

$$U = \begin{bmatrix} \frac{1}{3} & 0 & \frac{2\sqrt{2}}{3} \\ 0 & -1 & 0 \\ \frac{2\sqrt{2}}{3} & 0 & -\frac{1}{3} \end{bmatrix} \tag{11}$$

And

$$\sigma^{(\mathrm{NV}\,[\bar{1}\bar{1}1]\mathrm{frame})} = \overbrace{\begin{bmatrix} 0 & 0 & 0 \\ 0 & 0 & 0 \\ 0 & 0 & \frac{m}{9} \end{bmatrix}}^{\sigma_{\parallel}} + \overbrace{\begin{bmatrix} \frac{8m}{9} & 0 & -\frac{2\sqrt{2}m}{9} \\ 0 & 0 & 0 \\ -\frac{2\sqrt{2}m}{9} & 0 & 0 \end{bmatrix}}^{\sigma_{\perp}} \tag{12}$$

3.3 Stress susceptibility parameters

In the work of Barson et al.[13], the stress susceptibility parameters in the crystal frame are $a_1 = 4.86, a_2 = -3.7, b = -2.3,$ and $c = 3.5$ in units of MHz /GPa . And $\mathbf{\Pi} = (\Pi_x,\ \Pi_y,\ \Pi_z)$ in crystal frame ($\hat{\mathrm{X}} \parallel [100],\ \hat{\mathrm{Y}} \parallel [010], \hat{\mathrm{Z}} \parallel [001]$) is shown as:

$$\Pi_x{}^{(\text{crystal frame})} = b(2\sigma_{ZZ} - \sigma_{XX} - \sigma_{YY}) + c(2\sigma_{XY} - \sigma_{YZ} - \sigma_{ZX}) \tag{13}$$

$$\Pi_y{}^{(\text{crystal frame})} = \sqrt{3}[b(\sigma_{XX} - \sigma_{YY}) + c(\sigma_{YZ} - \sigma_{ZX})] \tag{14}$$

$$\Pi_z{}^{(\text{crystal frame})} = a_1(\sigma_{XX} + \sigma_{YY} + \sigma_{ZZ}) + 2a_2(\sigma_{YZ} + \sigma_{ZX} + \sigma_{XY}) \tag{15}$$

The transformation of supplementary Eq. (13)-(15) from the crystal frame to the NV frame can be done by the coordinate transformation of the stress tensor :

$$\sigma_{\{xyz\}} = R\sigma_{\{XYZ\}}R^{\mathrm{T}} \tag{16}$$

Where

$$R = \begin{bmatrix} -\frac{1}{\sqrt{6}} & -\frac{1}{\sqrt{6}} & \sqrt{\frac{2}{3}} \\ \frac{1}{\sqrt{2}} & -\frac{1}{\sqrt{2}} & 0 \\ \frac{1}{\sqrt{3}} & \frac{1}{\sqrt{3}} & \frac{1}{\sqrt{3}} \end{bmatrix} \tag{17}$$

And supplementary Eq. (13)-(15) can be written in the NV frame as:

$$\Pi_x = (-b - c)\big(\sigma_{yy} - \sigma_{xx}\big) + (\sqrt{2}b - \frac{\sqrt{2}}{2}c)2\sigma_{xz} \tag{18}$$

$$\Pi_y = (-b - c)2\sigma_{xy} + (\sqrt{2}b - \frac{\sqrt{2}}{2}c)2\sigma_{yz} \tag{19}$$

$$\Pi_z = (a_1 - a_2)\big(\sigma_{xx} + \sigma_{yy}\big) + (a_1 + 2a_2)\sigma_{zz} \tag{20}$$

Comparing supplementary Eq. (13)-(15) with (18)-(20), we obtain the relationship

between the parameters in different frames:

$$\alpha_1 = a_1 - a_2$$

$$\beta_1 = a_1 + 2a_2$$

$$\alpha_2 = -b - c$$

$$\beta_2 = \sqrt{2}b - \frac{\sqrt{2}}{2}c$$

And the stress susceptibility parameters in the NV frame are $\alpha_1 = 8.56, \beta_1 = -2.54, \alpha_2 = -1.2,$ and $\beta_2 = -5.73$ in units of $\mathrm{MHz/GPa}$.

3.4 Shift of the zero-field splitting D_s with stress.

The pressure P dependence of the zero-field splitting shift $D_s(P)$ can be expressed as:

$$D_s(P) = \alpha_1\left[\sigma_{xx}(P) + \sigma_{yy}(P)\right] + \beta_1\sigma_{zz}(P) \tag{21}$$

where $\alpha_1 = 8.56,$ and $\beta_1 = -2.54$ in units of $\mathrm{MHz/GPa}$ are the stress susceptibility parameters. Obviously, the negative value of β_1 implies that an increase in the stress component along the NV direction $\sigma_{zz}(P)$ leads to a decrease in the zero-field shift, indicating a slowing down effect on the shift of the zero-field splitting.

For diamond particles inside the pressure chamber, like in the work of Doherty et al[15], the stress environment is mainly hydrostatic:

$$\sigma_{xx}(P) = \sigma_{yy}(P) = \sigma_{zz}(P) = P \tag{22}$$

so that the zero-field splitting shift is shown as:

$$D_s(P)^{\text{hydrostatic pressure}} = (2\alpha_1 + \beta_1)P \tag{23}$$

where the linear shift is about$(2\alpha_1 + \beta_1)$=14.58 $\mathrm{MHz/GPa}$ in hydrostatic pressure.

In our work, the NV center is perpendicular to the surface in the (111)-cut anvil. The stress tensor can be expressed as:

$$\overbrace{\begin{bmatrix} \eta P & 0 & 0 \\ 0 & \eta P & 0 \\ 0 & 0 & \eta P \end{bmatrix}}^{\text{hydrostatic pressure } \rho} + \overbrace{\begin{bmatrix} 0 & 0 & 0 \\ 0 & 0 & 0 \\ 0 & 0 & (1-\eta)P \end{bmatrix}}^{\text{uniaxial stress } \sigma} \tag{24}$$

Where the coefficient η is 0.61 using a simplified model of a semi-infinite anvil.[14]

So the zero-field splitting shift is shown as:

$$D_s(P)^{\text{anvil}} = (2\eta\alpha_1 + \beta_1)P \tag{25}$$

The linear shift is about $(2\eta\alpha_1 + \beta_1)$=7.9 MHz/GPa in the (111)-cut anvil, which is close to the linear shift 7.24 MHz/GPa in Fig. 2b.

3.5 Peak intensity asymmetry in ODMR

In our study, we utilize Continuous-wave (CW) optically detected magnetic resonance, where the intensity of spectral peaks displays an asymmetry. This asymmetry is attributed to the asymmetry of the effective microwave driving strength. As shown in Fig. 2a, there exists a disparity in the microwave driving strength between the f^+ and f^- peaks at zero magnetic field. This difference is directly related to the transverse term of the effective electric field Π_x and Π_y, which is associated with the proportion of the stress component $\sigma_\perp: \{(\sigma_{yy} - \sigma_{xx}), \sigma_{xz}, \sigma_{xy}, \sigma_{yz}\}$). The theoretical explanation and experimental validation are outlined below.

The ODMR is induced by an oscillating magnetic field generated by microwave radiation. In our experiments, the microwave fields are linearly polarized. This microwave interaction part of the Hamiltonian can be represented as:

$$H_{\text{mw}} = \gamma_{\text{e}} B_1 cos\omega t\left(cos\varphi_{\text{mw}} S_x + sin\varphi_{\text{mw}} S_y\right) \tag{26}$$

where $B_1$is the strength of the transverse part microwave field; ω is the microwave frequency; φ_{mw} is the angle between the transverse part of the microwave field and the $\hat{\text{x}}$-axis.

Transforming to the rotated frame $U = e^{-i\omega t S_z^2}$ and ignoring the high frequency oscillation term $e^{-2i\omega t}$ (the rotating wave approximation), the Hamiltonian of NV center can be written as:

$$\overbrace{\begin{bmatrix} D_0+\Pi_z+\Delta_B & 0 & -\Pi_x-i\Pi_y \\ 0 & 0 & 0 \\ -\Pi_x+i\Pi_y & 0 & D_0+\Pi_z-\Delta_B \end{bmatrix}}^{H_0=H_D+H_B+H_S}+\overbrace{\begin{bmatrix} 0 & \frac{\gamma_e B_1 e^{-i\varphi_{\mathrm{mw}}}}{2\sqrt{2}} & 0 \\ \frac{\gamma_e B_1 e^{i\varphi_{\mathrm{mw}}}}{2\sqrt{2}} & 0 & \frac{\gamma_e B_1 e^{-i\varphi_{\mathrm{mw}}}}{2\sqrt{2}} \\ 0 & \frac{\gamma_e B_1 e^{i\varphi_{\mathrm{mw}}}}{2\sqrt{2}} & 0 \end{bmatrix}}^{H_{\mathrm{mw}}}-\omega {S_z}^2 \quad (27)$$

where the resonance condition is satisfied:

$$\omega=f^{\pm}=D_0+\Pi_z\pm\sqrt{{\Pi_x}^2+{\Pi_y}^2+{\Delta_B}^2} \quad (28)$$

The transverse term of the effective electric field (Π_x, Π_y) with Δ_B mixes the eigenstates of H_0 from $\{|0\rangle, |\pm1\rangle\}$ to a new basis $\{|0\rangle, |\pm\rangle\}$. In order to conveniently represent the new eigenstates, we set:

$$\begin{cases} \Pi_x=\Delta\, sin\theta\, cos\varphi \\ \Pi_y=\Delta\, sin\theta\, sin\varphi \\ \Delta_B=\Delta\, cos\theta \end{cases} \quad (29)$$

Here $0\le\theta\le\pi/2; -\pi\le\varphi\le\pi$. And φ is the angle between the transverse part of the effective electric field and the $\hat{x}$-axis.

The new eigenstates of H_0 can be express as:

$$\begin{cases} |+\rangle=-e^{i\varphi}\cos\frac{\theta}{2}|+1\rangle+\sin\frac{\theta}{2}|-1\rangle \\ |0\rangle=|0\rangle \\ |-\rangle=e^{i\varphi}\sin\frac{\theta}{2}|+1\rangle+\cos\frac{\theta}{2}|-1\rangle \end{cases} \quad (30)$$

The $|0\rangle\leftrightarrow|\pm\rangle$ resonance microwave driving strength $\Omega_{0\pm}$ is related to the transition matrix element:

$$\Omega_{0+}=2\langle 0|H_{\mathrm{mw}}|+\rangle=\left|\frac{B_1\gamma_e}{\sqrt{2}}\left(-e^{i(\varphi+2\varphi_{\mathrm{mw}})}\cos\frac{\theta}{2}+\sin\frac{\theta}{2}\right)\right| \quad (31)$$

$$\Omega_{0-}=2\langle 0|H_{\mathrm{mw}}|-\rangle=\left|\frac{B_1\gamma_e}{\sqrt{2}}\left(e^{i(\varphi+2\varphi_{\mathrm{mw}})}\sin\frac{\theta}{2}+\cos\frac{\theta}{2}\right)\right| \quad (32)$$

From the above expressions, $\Omega_{0\pm}$ is determined by B_1, φ_{mw}, φ, and, Δ_B

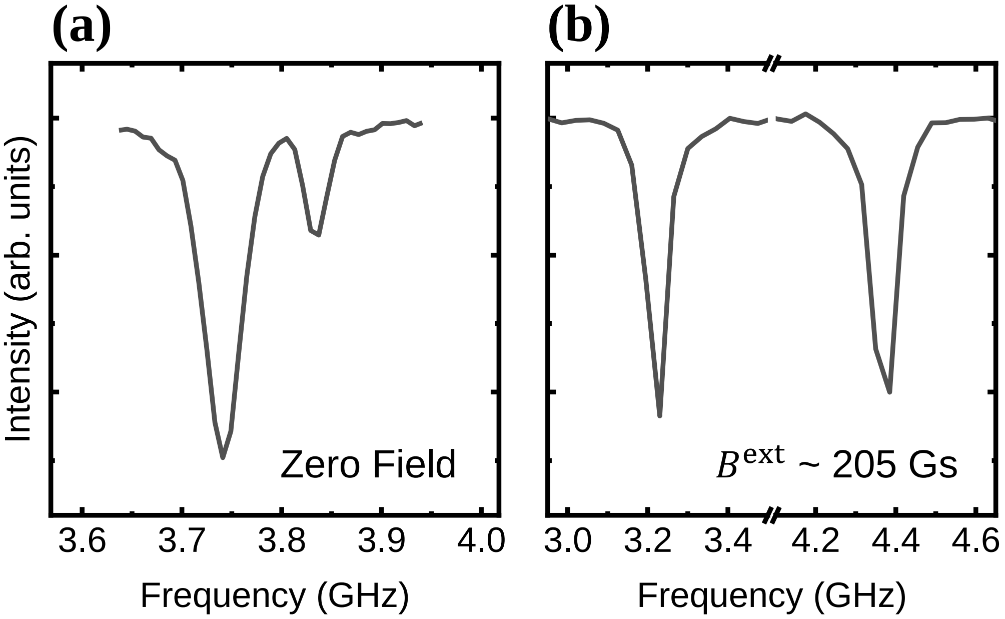

Supplementary Fig. 4. **Correlation of CW ODMR intensity with magnetic field at 130 GPa.** (a)(b) the ODMR data get in zero field and 205 G magnetic field.

In zero magnetic field situation (Δ_B= 0, $\theta = \arccos\left(\frac{\Delta_B}{\Delta}\right) = \frac{1}{2}\pi$), the effective driving strength is:

$$\Omega_{0+} = \left|\frac{B_1\,\gamma_{\mathrm{e}}}{2}\left(-e^{i(\varphi+2\varphi_{\mathrm{mw}})} + 1\right)\right| \tag{33}$$

$$\Omega_{0-} = \left|\frac{B_1\,\gamma_{\mathrm{e}}}{2}\left(e^{i(\varphi+2\varphi_{\mathrm{mw}})} + 1\right)\right| \tag{34}$$

Ω_{0+} and Ω_{0-} are thus different and correlated with the φ and φ_{mw}. This difference leads to differences in the intensity of the two peaks $(f^{\pm})$, e.g., when both transverse part of the microwave field and the effective electric field is along the $\hat{\mathrm{x}}$-axis ($\varphi_{\mathrm{mw}} \to 0, \varphi \to 0$), the $\Omega_{0+} \to 0, \Omega_{0-} \to B_1\,\gamma_{\mathrm{e}}$, resulting in the left peak being significantly stronger than the right peak, as shown in Supplementary Fig. 4a and Fig. 2a.

In the presence of a sufficiently strong external magnetic field ($\Delta_B \gg \Delta_{\sigma_\perp}$, $\theta = \arccos\left(\frac{\Delta_B}{\Delta}\right) \to 0$), the state mixing due to $\sigma_\perp$ is suppressed. The effective driving strength is modified as:

$$\Omega_{0+} = \Omega_{0-} \to \left|\frac{B_1\,\gamma_{\mathrm{e}}}{\sqrt{2}}\right| \tag{35}$$

This can lead to the same intensity of the two peaks $(f^{\pm})$. (as shown in Supplementary Fig. 4b)

4 Supplementary Note 2: Impact of uniaxial stress on [111] and non-[111] NV centers.

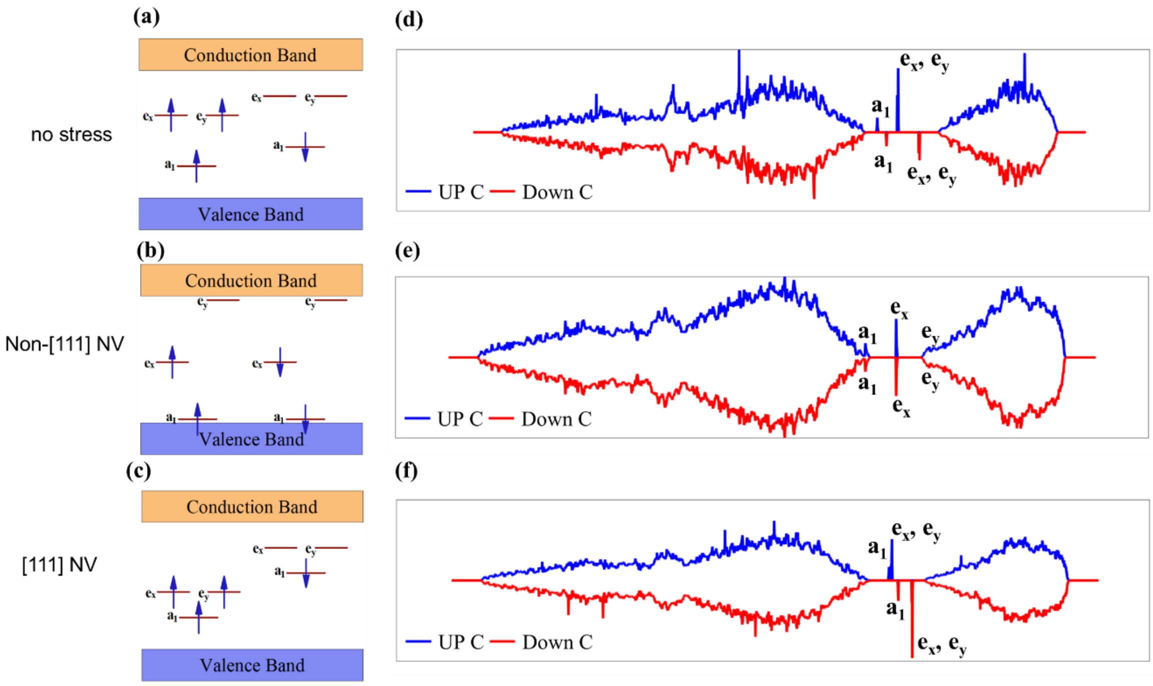

Supplementary Fig. 5. First-principles calculation of the NV centers in 132 GPa uniaxial stress along the diamond [111] direction. (a)-(c) The electronic ground state levels of NV center with no stress, NV centers along $[\mathbf{1}\bar{\mathbf{1}}\bar{\mathbf{1}}]$, $[\bar{\mathbf{1}}\mathbf{1}\bar{\mathbf{1}}]$, and $[\bar{\mathbf{1}}\bar{\mathbf{1}}\mathbf{1}]$ direction(non-[111] NV), NV centers along [111] direction([111] NV), respectively. (d)-(f) The PDOS (Projected Density of States) spectra for no stress, non-[111] NV, and [111] NV, respectively. The PDOSs are calculated using the PBE functional. In the PDOS spectra, all energies are relative to the Fermi level at 0 eV. The individual PDOS spectra show projections for the C atoms.

To study the impact of uniaxial stress on the sensors, we used VASP to carry out first-principles calculation of the electronic ground state levels of the NV center in stress environment. Constructed from linear combinations of the dangling sp^3 orbitals of three nearest-neighbor carbons and one nitrogen atom, the molecular orbitals configuration of an NV center consists of four states: $a_1(1)$, $a_1(2)$ (of a_1 symmetry), and e_x, e_y (of e symmetry) [16]. Since $a_1(1)$ is in the valance band and is always fully occupied,

it is not shown in Supplementary Fig. 5, where a_1 represents $a_1(2)$.

Under ambient pressure, the NV center exhibits a C_{3V} symmetry, thus e_x, e_y are degenerate and occupied by two electrons of the same spin orientation (Supplementary Fig. 5a and d), which form a spin triplet (S = 1). When the uniaxial stress is applied along the [111] direction, the C_{3V} symmetry would be broken for the non-[111] NV centers ($[1\bar{1}\bar{1}]$, $[\bar{1}1\bar{1}]$, and $[\bar{1}\bar{1}1]$), accompanied by a shift in energy levels and a rearrangement of electrons, resulting in a single state (S=0) at uniaxial stress 132 GPa (Supplementary Fig. 5b and e). In contrast, for the [111] NV centers with the same direction of the uniaxial stress, the spin-triplet configuration is maintained (Supplementary Fig. 5 c and f).

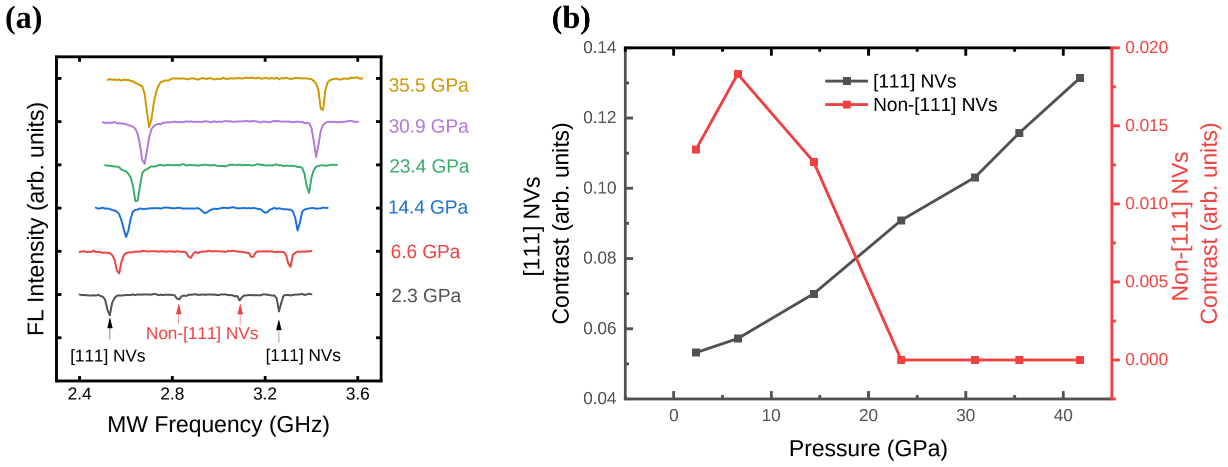

Supplementary Fig. 6. Effect of stress on ODMR spectra in (111)-cut diamond anvil. (a) Variation of ODMR spectra with rising pressure, where stand peaks of [111] NV centers (black arrow) and peaks of non-[111] NV centers (red arrow). **(b)** Pressure dependence ODMR contrast for [111] NV centers (in black) and non-[111] NV centers (in red).

We observe that the fluorescence signals from both [111] centers and non-[111] oriented NV centers match well with the above predictions. We investigate the development of the ODMR spectrum and the signal contrast of [111] and non-[111] oriented NV centers as the pressure increases. To study the NV centers of different orientations separately, we apply a magnetic field of 144 G along the [111] orientation, which splits the ODMR spectrum of the NV centers ensemble into four peaks (Supplementary Fig. 6a, 2.3 GPa). The peaks indicated by black arrows belong to [111] NV centers, while the peaks indicated by red arrows belong to non-[111] NV centers.

For [111] NV centers, the presence of non-[111] NV centers contributes to background fluorescence signals that are approximately triple the intensity of the [111] NV centers. This background fluorescence thus limits the ODMR contrast of [111] NV centers to be maximally around ~8%, which has been observed in many other independent works.[17]

As the pressure increases, we observe an enhancement of the contrast of the [111] peaks and a generally opposite trend for the non-[111] NV centers (Supplementary Fig. 6b). As the pressure exceeds 23.4 GPa, the signal from non-[111] NV centers disappears, and the increasing trend of the contrast of the [111] NV centers does not stop. The contrast of [111] NV centers increases to around 30% (Fig. 2f) at 130 GPa, which is the optimal signal contrast achievable for a single NV center at ambient pressures. We suspect that fluorescence quenching may occur for non-[111] NV centers at high pressure and thus increase the ODMR contrast of the [111] NV centers to optimal.

5 Supplementary Note 3: Experiments with diamond particles loaded inside a high-pressure chamber

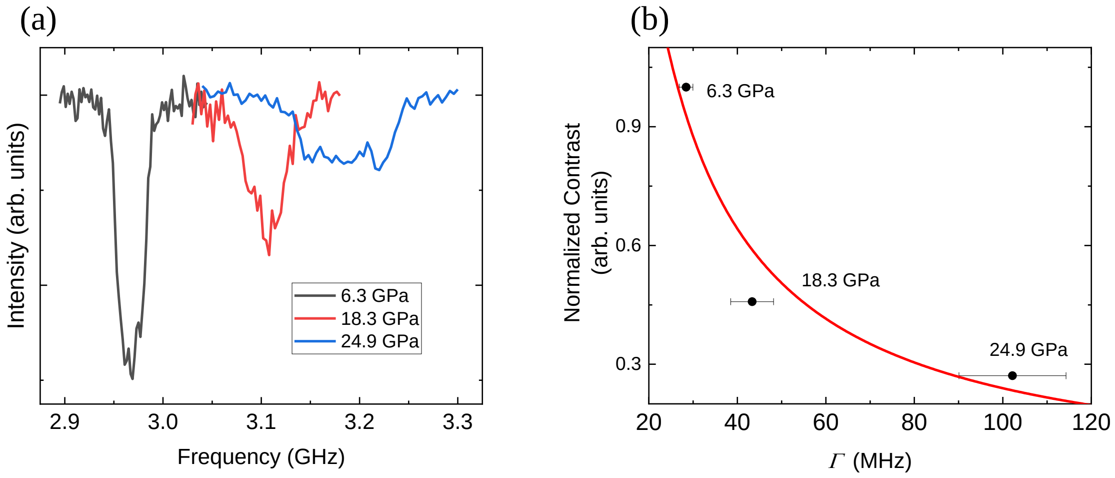

Supplementary Fig. 7. ODMR experiments with diamond particles loaded inside a high-pressure chamber. (a) The ODMR spectral lines with elevated pressure. (b) spectral line width ($\boldsymbol{\Gamma}$) dependence of normalized contrast. The black dots show the data from (a), and the experimental data are fitted by function $\mathbf{y} = \boldsymbol{a}\mathbf{x}^{\boldsymbol{b}}$ and plotted by red solid lines. The fitting parameters $\boldsymbol{b} = -\mathbf{1.08} \pm \mathbf{0.34}$, which indicate the relationship between line width and contrast approximates an inverse proportional correlation. The error bars in (b) correspond to a 95% confidence interval.

To better understand the limitations of using NV in a pressure chamber, we performed experiments with diamond particles (1-2 μm from FND Biotech, INC.) loaded inside a high-pressure chamber. Achieving an ideal homogeneous hydrostatic condition in a high-pressure chamber poses challenges due to microscopic stresses introduced by imperfect sample crystals and assembly. Especially under extreme pressures, utilizing solid PTM becomes somewhat inevitable. It's important to note that solid PTM inherently differs from fluid PTM as they exhibit shear stress and stress inhomogeneity up to their shear strength limit[18]. As shown in Supplementary Fig. 7, the experimental results demonstrate that the presence of the shear stress and stress inhomogeneity leads to a sharp broadening of the spectral lines and consequent contrast degradation with pressure. Based on the current trend of spectral line changes, the

continuous broadening with pressure causes the ODMR contrast to drop dramatically, limiting the sensitivity and accuracy of measurements.

6 Supplementary Note 4: Magnetic signals and error analysis

6.1 Extracting the magnetic field B_z^{mag} induced by magnetite from the Reference NV.

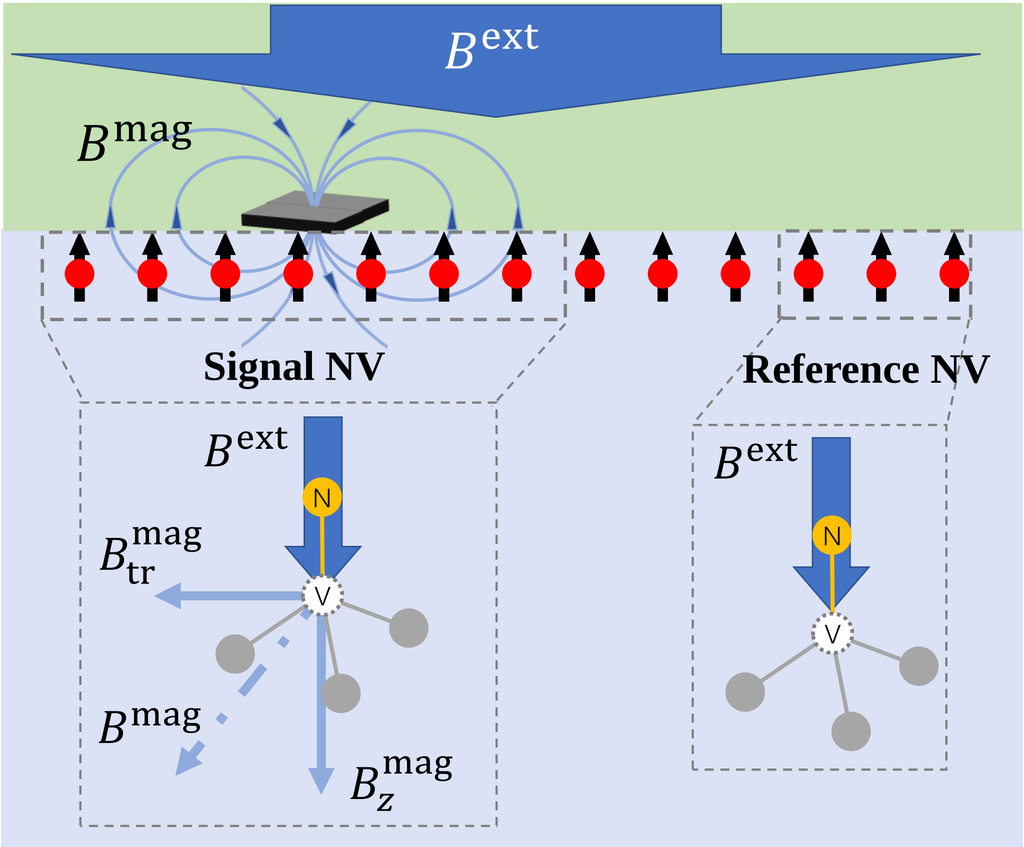

Supplementary Fig. 8. Schematic drawing of signal NV and reference NV. The external magnetic field is the $\vec{\boldsymbol{B}}^{\mathbf{ext}}$ along the NV axis. The NV center near magnetite (Signal NV) is affected by the external magnetic field $\vec{\boldsymbol{B}}^{\mathbf{ext}}$ and the magnetite field $\vec{\boldsymbol{B}}^{\mathbf{mag}}$, and $\vec{\boldsymbol{B}}^{\mathbf{mag}}$ can be decomposed into $\boldsymbol{B}_{\mathbf{tr}}^{\mathbf{mag}}$ perpendicular to the NV axis and $\boldsymbol{B}_{z}^{\mathbf{mag}}$ along the NV axis. And the NV center far away from the magnetite (Reference NV) is affected only by $\vec{\boldsymbol{B}}^{\mathbf{ext}}$, which is used as a reference for the calibration of the external magnetic field in the experiments.

The images of the magnetic field are obtained from the optically detected magnetic resonance (ODMR) spectrum of the NV centers. As shown in Fig. 1c, under magnetic field and stress, the energy levels of the NV center change, resulting in shifting and splitting of the ODMR peaks.

The transverse component of the magnetic field, denoted as $B_{\mathrm{tr}}^{\mathrm{mag}}$, is assumed to be sufficiently small than the external magnetic field B^{ext}. In the weak magnetic field approximation, the half Zeeman splitting Δ_B can be approximated by the magnetic field component along the NV center axis B_z as:

$$\Delta_B = \gamma_{\mathrm{e}} B_z \tag{36}$$

The splitting of the ODMR peaks can be expressed as:

$$2\Delta = 2\sqrt{{\Delta_B}^2 + {\Delta_{\sigma_\perp}}^2} \tag{37}$$

Since $\Delta_B \gg \Delta_{\sigma_\perp}$, we can ignore the existence of $\Delta_{\sigma_\perp}$. Supplementary Eq. (37) simplifies to:

$$\Delta \cong \Delta_B \tag{38}$$

As shown in Supplementary Fig. 8, the external magnetic field B^{ext} needs to be subtracted from the total magnetic field when measuring the magnetite magnetic field via Signal NV. A reference NV, which is located farther away from the magnetite, remains unaffected by the magnetic field generated by the magnetite and is used to calibrate the external magnetic field. The magnetic field component B_z^{mag} induced by magnetite can be extracted from the external magnetic field using the following relation:

$$B_z^{\mathrm{mag}} = \frac{\Delta_{\mathrm{sig}} - \Delta_{\mathrm{ref}}}{\gamma_{\mathrm{e}}} \tag{39}$$

Here, $\Delta_{\mathrm{sig/ref}} = (f_{\mathrm{sig/ref}}^{+} - f_{\mathrm{sig/ref}}^{-})/2$ are the half splitting of the ODMR peaks in signal NV and reference NV acquired in the experiment. The frequencies $f_{\mathrm{sig/ref}}^{\pm}$ are the transition frequencies of $|0\rangle$ to $|\pm\rangle$ obtained by fitting the full ODMR spectrum peaks.

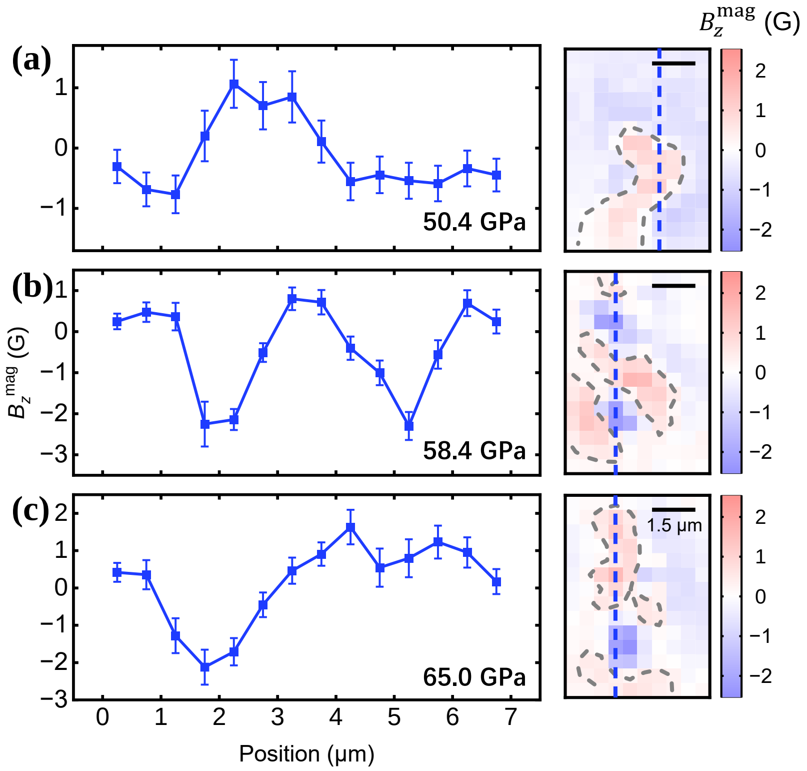

Supplementary Fig. 9. Demonstration of magnetic imaging data with error bar. (a), (b), and (c) show the imaging results at 50.4, 58.4, and 65 GPa, respectively. The data marked by the blue dashed line on the right are shown with error bars on the left. The error bars correspond to a 95% confidence interval.

We can obtain the error of B_z^{mag} by error propagation:

$$\mathrm{ero}B_z^{\mathrm{mag}} = \frac{\sqrt{\mathrm{ero}{f_{\mathrm{sig}}^{-}}^2 + \mathrm{ero}{f_{\mathrm{sig}}^{+}}^2 + \mathrm{ero}{f_{\mathrm{ref}}^{-}}^2 + \mathrm{ero}{f_{\mathrm{ref}}^{+}}^2}}{2\gamma_{\mathrm{e}}} \tag{40}$$

$\mathrm{ero}f_{\mathrm{sig/ref}}^{\pm}$ represents the fitting errors of the transition frequencies in fitting the corresponding spectrum. The data points in Fig. 4 a, b and e to i are obtained by supplementary Eq. (39), and the error bars corresponding to a 95% confidence interval are obtained by supplementary Eq. (40). Supplementary Fig. 9 shows the B_z^{mag} data and its error bars.

6.2 Uniformity of the external magnetic field

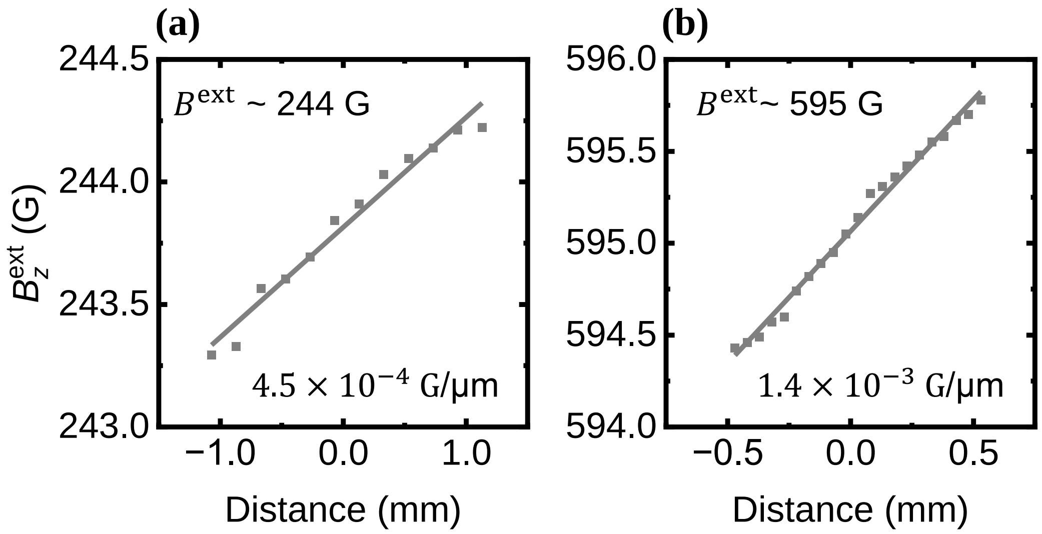

Supplementary Fig. 10. Testing of magnetic field gradients. (a) and (b) are the spatial distributions of the magnetic field when the magnetic field strengths of 244 G and 595 G are tested by a gaussmeter, respectively. By linearly fitting the data, the gradient of the magnetic field in space is $\mathbf{1.4 \times 10^{-3}}$ **G/μm**@595 G and $\mathbf{4.5 \times 10^{-4}}$ **G/μm** @244 G

To ensure the consistency of the external magnetic field at Signal NV and Reference NV, we test the magnetic field gradient in space of the NdFeB magnets used to apply the external magnetic field. As shown in Supplementary Fig. 10, the gradient of the magnetic field in space is approximately 1.4×10^{-3} G/μm @600 G and 4.5×10^{-4} G/μm @244 G. The error from external magnetic field uniformity is thus much less than 0.02 G and can be ignored.

6.3 Relative error for weak magnetic field approximation $(\Delta^{sim} - \Delta)/\Delta$

In this section, we aim to quantify the error in measuring the magnetic field of magnetite using the weak magnetic field approximation. To do so, we simulate the induced splitting of the ODMR peaks $2\Delta^{sim}$ by numerically calculating the eigenvalues of the following Hamiltonian (supplementary Eq. (2)):

$$H = \begin{bmatrix} D_0 + D_s + \gamma_e B_z & \frac{\gamma_e}{\sqrt{2}}(B_x - iB_y) & \Delta_{\sigma\perp} \\ \frac{\gamma_e}{\sqrt{2}}(B_x + iB_y) & 0 & \frac{\gamma_e}{\sqrt{2}}(B_x - iB_y) \\ \Delta_{\sigma\perp} & \frac{\gamma_e}{\sqrt{2}}(B_x + iB_y) & D_0 + D_s - \gamma_e B_z \end{bmatrix} \quad (41)$$

On the other hand, for weak magnetic field approximation, the splitting of ODMR peaks is given by 2Δ, as expressed in supplementary Eq. (37). By comparing the simulated splitting of the ODMR peaks with the splitting obtained from the weak magnetic field approximation, we can determine the magnitude of the error and evaluate the validity of the approximation in accurately measuring the magnetic field of magnetite.

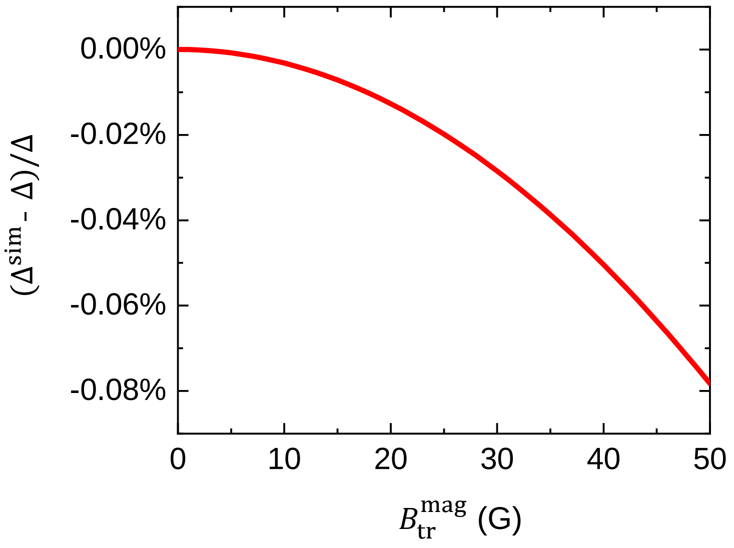

Supplementary Fig. 11. Numerical estimation of relative error for the weak magnetic field approximation. $\boldsymbol{B_{\mathrm{tr}}^{\mathrm{mag}}}$ dependence of the weak magnetic field approximation relative error $(\Delta^{\mathrm{sim}} - \Delta)/\Delta$.

In the experiment of the magnetic image at pressure ~60 GPa, we set stress evolution terms $D_s = 440$ MHz and $\Delta_{\sigma\perp} = 20$ MHz for calculation.

For the longitudinal component of the magnetic field:

$$B_z = B^{\mathrm{ext}} + B_z^{\mathrm{mag}} \quad (42)$$

Where the external magnetic field B^{ext} is about 240 G. and the B_z^{mag} is about -20 G to 20 G. So, we set $B_z = 240$ G for calculation.

For the transverse component of the magnetic field:

$$B_x = B_{\mathrm{tr}}^{\mathrm{mag}} \cos\emptyset \tag{43}$$

$$B_y = B_{\mathrm{tr}}^{\mathrm{mag}} \sin\emptyset \tag{44}$$

$\emptyset$ is the azimuthal angle in the transverse magnetic field plane and its value has little effect on the numerical results (we set $\emptyset = 0$ in the calculation). And the magnitude of the transverse field $B_{\mathrm{tr}}^{\mathrm{mag}}$ should be comparable to the longitudinal field $B_{\mathrm{z}}^{\mathrm{mag}}$(3-20 G). Supplementary Fig. 11 shows the variation of the relative error with $B_{\mathrm{tr}}^{\mathrm{mag}}$, and the relative error should be much less than 0.1%. In Fig. 4f, assuming the presence of a 40 G transverse field, the measured magnetic field B_z^{mag} =18 G is only deviated by ~0.1 G.

6.4 Relative deviation for $\Delta_B \gg \Delta_{\sigma_\perp}$ condition.

In simplifying supplementary Eq. (37) to (38), we have assumed the condition of $\Delta_B \gg \Delta_{\sigma_\perp}$. However, this assumption may introduce a deviation in the calculated magnetic field measurement. The magnitude of this deviation depends on the relative values of Δ_B and the stress splitting term $\Delta_{\sigma_\perp}$.

For the reference NV, we observe a splitting $2\Delta_{ref}$ given by supplementary Eq. (37):

$$2\Delta_{\mathrm{ref}} = 2\sqrt{\Delta_B^{\mathrm{ext}^2} + \Delta_{\sigma_\perp}{}^2}$$

For the signal NV, we observe a splitting $2\Delta_{sig}$ given by supplementary Eq. (37):

$$2\Delta_{\mathrm{sig}} = 2\sqrt{\left(\Delta_B^{\mathrm{ext}} + \Delta_B^{\mathrm{mag}}\right)^2 + \left(\Delta_{\sigma_\perp} + \delta * \Delta_{\sigma_\perp}\right)^2} \tag{45}$$

Where $\Delta_B^{\mathrm{ext}} = \gamma_{\mathrm{e}} B^{\mathrm{ext}}$ and $\Delta_B^{\mathrm{mag}} = \gamma_{\mathrm{e}} B_z^{\mathrm{mag}}$. $\delta * \Delta_{\sigma_\perp}$ is the stress splitting term due to spatial stress differences between reference and signal NV.

The third-order series expansion of $\frac{\Delta_{\mathrm{sig}} - \Delta_{\mathrm{ref}}}{\gamma_{\mathrm{e}}}$ can be written as:

$$\frac{\Delta_{\mathrm{sig}} - \Delta_{\mathrm{ref}}}{\gamma_{\mathrm{e}}} = B_z^{\mathrm{mag}} + \overbrace{\frac{1}{2\gamma_{\mathrm{e}}} \Delta_B^{\mathrm{ext}} \left[\frac{\Delta_B^{\mathrm{ext}}(1+\delta)^2}{\Delta_B^{\mathrm{ext}} + \Delta_B^{\mathrm{mag}}} - 1\right] \left(\frac{\Delta_{\sigma_\perp}}{\Delta_B^{\mathrm{ext}}}\right)^2}^{Dev} + \frac{1}{\gamma_{\mathrm{e}}} O^4 \left(\frac{\Delta_{\sigma_\perp}}{\Delta_B^{\mathrm{ext}}}\right) \tag{46}$$

The second-order term is the magnetic field deviation Dev when using the $\Delta = \Delta_B$ approximation. When the magnetic field generated by the magnetite is extremely weak ($B_z^{\text{mag}} \to 0$) in high pressure, Dev can be expressed as:

$$\text{Dev} = \frac{{\Delta_{\sigma_\perp}}^2}{\Delta_B^{\text{ext}}} \frac{\delta(\delta+2)}{2\gamma_{\text{e}}} \tag{47}$$

	B^{ext}	Dev_{max}
Fig. 4c Exp1	130 G	0.22 G @80 GPa($\Delta_{\sigma_\perp}$~25 MHz)
Fig. 4c Exp2	570 G	0.2 G @130 GPa($\Delta_{\sigma_\perp}$~50 MHz)
Fig. 4b Exp2	240 G	0.12 G @80 GPa($\Delta_{\sigma_\perp}$~25 MHz)
Fig. 4f to j	240 G	0.07 G @65 GPa($\Delta_{\sigma_\perp}$~20 MHz)

Supplementary Table 2. The maximum deviation Dev_{max} due to $\Delta = \Delta_B$ approximation in experiments

The stress splitting term $\Delta_{\sigma_\perp}$ was tested randomly on the anvil and the standard deviation was about 16%. We take twice the standard deviation as the difference in stress splitting term ($\delta = 32\%$) to calculate the maximum deviation of the magnetic field Dev_{max} due to approximation. Table. S2 shows the maximum deviations of the magnetic field at the highest pressure point, which are much smaller than 0.5 G and can be ignored.

Although not happening in this experiment, it is worth noting that when the external magnetic field is too small or the stress splitting term is too large, it may not be sufficient to satisfy the $\Delta_B \gg \Delta_{\sigma_\perp}$ condition for magnetic field measurements, and measurement of the stress term in the environment is necessary. The sample's own magnetic field can interfere with the measurement of the stress term, which can be calibrated by adjusting the temperature above the Curie/Néel temperature to "turn off" the sample's magnetic field.

Considering the adverse effects of the $\sigma_\perp$ component on the zero-filed application of NV-based quantum sensing, it may be possible in the future to optimize the uniaxial pressure by employing an ideal (111)-cut anvil or using gas pressure-transmitting media

such as Ne or Ar. This optimization may further suppress the generation of $\Delta_{\sigma_\perp}$.

6.5 Analysis and discuss for ODMR spectra linewidth broadening Γ^{mag}

The ODMR spectra linewidth broadening Γ^{mag} is obtained from:

$$\Gamma^{\mathrm{mag}} = \Gamma^{\mathrm{sig}} - \Gamma^{\mathrm{ref}} \tag{48}$$

Γ^{sig} and Γ^{ref} are the linewidth of the ODMR peaks in signal NV (at NV_i) and reference NV (in PTM), respectively. And the error of Γ^{mag} is obtained by error propagation:

$$\mathrm{ero}\Gamma^{\mathrm{mag}} = \sqrt{\mathrm{ero}\Gamma^{\mathrm{sig}^2} + \mathrm{ero}\Gamma^{\mathrm{ref}^2}} \tag{49}$$

$ero\Gamma^{mag}$ corresponding to the fitting errors of the linewidth of the ODMR peaks in Signal NV and Reference NV in spectrum peak fitting. The data points in Fig. 4d are obtained by supplementary Eq. (48), and the error bars corresponding to a 95% confidence interval are obtained by supplementary Eq. (49).

The distinct mechanical properties of magnetite compared to the pressure-transmitting medium (PTM) may lead to differences in the linewidth broadening between the NV_i (Signal NV) and Reference NV. Typically, this difference increases with higher pressure. To assess the impact of these varying mechanical properties, we examine the ODMR linewidth difference ($\Gamma^{\mathrm{sig}} - \Gamma^{\mathrm{ref}}$) in the pressure range above 70 GPa (Supplementary Fig. 12). Notably, at these high pressures, magnetite exhibits paramagnetic behavior and lacks macroscopic magnetization, implying that the linewidth broadening is primarily due to environmental stress gradients.

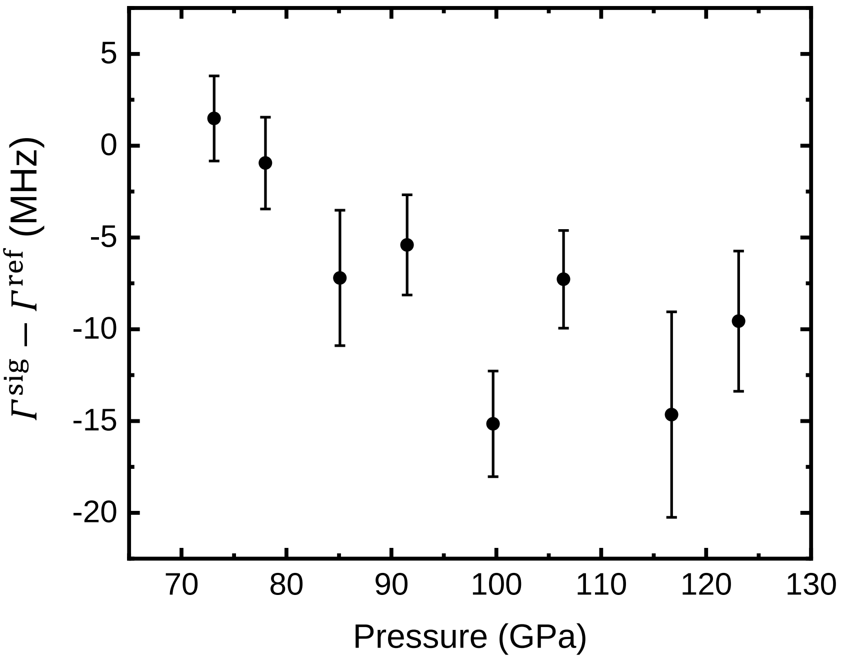

Supplementary Fig. 12. Pressure dependence of the linewidth differences $\boldsymbol{\Gamma}^{\mathbf{sig}} - \boldsymbol{\Gamma}^{\mathbf{ref}}$. The black dots show the data from 70 GPa to 130 GPa.The error bars correspond to a 95% confidence interval.

As depicted in the Supplementary Fig. 12, the linewidth broadening exhibits differences of about 10-20 MHz only when the pressure exceeds 100 GPa. Interestingly, under the magnetite, the linewidth is even smaller. This observation suggests that the linewidth caused by the mechanical properties difference of magnetite does not significantly contribute to the linewidth broadening Γ^{mag} in the range of hundreds of megahertz or greater.

The linewidth broadening of the ODMR spectrum in NV_i is attributed not only to inhomogeneous stresses but also to strong spatial magnetic field gradients and potential magnetic fluctuations near the surface of magnetite. Further experimental evidence is needed to elucidate the contributions of both the "magnetic fluctuations in time domain" effect and the "magnetic field variation in space" effect to the spectral line broadening beneath the sample. However, the linewidth broadening of the ODMR spectrum can still serve as a basis for assessing whether magnetite exhibits macroscopic magnetism, alongside the detection of stray magnetic fields.

Notably, small-scale thermal fluctuation behaviors of magnetic domain walls are commonplace in magnetic materials. These fluctuations have been observed in various microscopic imaging techniques as blurry regions or evidenced by changes in stripe position or density between individual image line scans [19-21]. High temporal resolution techniques have also been reported to observe domain wall fluctuations in real-time in ferromagnetic films[22]. Additionally, linewidth broadening of the ESR spectrum due to magnetic fluctuations near magnetic domains has been observed in scanning single-NV microscopy [23].

At present, the mechanism behind magnetic fluctuations is still under investigation, with research methods being quite limited. It may be associated with the competition between short-range strong exchange interactions and long-range weak antiferromagnetic dipole interactions. While our current work may not fully eliminate the impact of magnetic field gradients, further advancements in single NV technology are anticipated to facilitate quantitative observations during pressure regulation, thereby aiding in mechanism research.

References in Supplementary

1 Dewaele, A., Datchi, F., Loubeyre, P. & Mezouar, M. High pressure--high temperature equations of state of neon and diamond. *Phys. Rev. B* **77**, 094106, doi:10.1103/PhysRevB.77.094106 (2008).

2 Akahama, Y. & Kawamura, H. Pressure calibration of diamond anvil Raman gauge to 310GPa. *J. Appl. Phys.* **100**, doi:10.1063/1.2335683 (2006).

3 Akahama, Y. & Kawamura, H. Raman study on the stress state of [111] diamond anvils at multimegabar pressure. *J. Appl. Phys.* **98**, doi:10.1063/1.2115098 (2005).

4 Hsieh, S. *et al.* Imaging stress and magnetism at high pressures using a nanoscale quantum sensor. *Science* **366**, 1349-1354, doi:10.1126/science.aaw4352 (2019).

5 Lesik, M. *et al.* Magnetic measurements on micrometer-sized samples under high pressure using designed NV centers. *Science* **366**, 1359-1362, doi:10.1126/science.aaw4329 (2019).

6 Yip, K. Y. *et al.* Measuring magnetic field texture in correlated electron systems under extreme conditions. *Science* **366**, 1355-1359, doi:10.1126/science.aaw4278 (2019).

7 Shang, Y. X. *et al.* Magnetic Sensing inside a Diamond Anvil Cell via Nitrogen-Vacancy Center Spins. *Chin. Phys. Lett.* **36**, doi:Artn 086201
10.1088/0256-307x/36/8/086201 (2019).

8 Doherty, M. W. *et al.* Electronic properties and metrology applications of the diamond NV- center under pressure. *Phys. Rev. Lett.* **112**, 047601, doi:10.1103/PhysRevLett.112.047601 (2014).

9 Dai, J.-H. *et al.* Optically Detected Magnetic Resonance of Diamond Nitrogen-Vacancy Centers under Megabar Pressures. *Chin. Phys. Lett.* **39**, 117601, doi:10.1088/0256-307X/39/11/117601 (2022).

10 Hilberer, A. *et al.* Enabling quantum sensing under extreme pressure: Nitrogen-vacancy magnetometry up to 130 GPa. *Phys. Rev. B* **107**, L220102, doi:10.1103/PhysRevB.107.L220102 (2023).

11 Wang, J. F. *et al.* Magnetic detection under high pressures using designed silicon vacancy centres in silicon carbide. *Nat. Mater.* **22**, 489-494, doi:10.1038/s41563-023-01477-5 (2023).

12 Liu, L. *et al.* Coherent Control and Magnetic Detection of Divacancy Spins in Silicon Carbide at High Pressures. *Nano Lett.* **22**, 9943-9950, doi:10.1021/acs.nanolett.2c03378 (2022).

13 Barson, M. S. J. *et al.* Nanomechanical Sensing Using Spins in Diamond. *Nano Lett.* **17**, 1496-1503, doi:10.1021/acs.nanolett.6b04544 (2017).

14 Ruoff, A. L., Luo, H. & Vohra, Y. K. The closing diamond anvil optical window in multimegabar research. *J. Appl. Phys.* **69**, 6413-6416, doi:10.1063/1.348845 (1991).

15 Doherty, M. W. *et al.* Electronic properties and metrology applications of the diamond NV- center under pressure. *Phys. Rev. Lett.* **112**, 047601, doi:10.1103/PhysRevLett.112.047601 (2014).

16 Gali, A., Fyta, M. & Kaxiras, E. Ab initio supercell calculations on nitrogen-vacancy center in diamond: Electronic structure and hyperfine tensors. *Phys. Rev. B* **77**, doi:ARTN 155206

10.1103/PhysRevB.77.155206 (2008).

17 Barry, J. F. *et al.* Sensitivity optimization for NV-diamond magnetometry. *Rev. Mod. Phys.* **92**, 015004, doi:10.1103/RevModPhys.92.015004 (2020).

18 Celeste, A., Borondics, F. & Capitani, F. Hydrostaticity of pressure-transmitting media for high pressure infrared spectroscopy. *High Pressure Research* **39**, 608-618, doi:10.1080/08957959.2019.1666844 (2019).

19 Portmann, O., Vaterlaus, A. & Pescia, D. An inverse transition of magnetic domain patterns in ultrathin films. *Nature* **422**, 701-704, doi:10.1038/nature01538 (2003).

20 Won, C. *et al.* Magnetic stripe melting at the spin reorientation transition in Fe / Ni / Cu(001). *Phys. Rev. B* **71**, 224429, doi:10.1103/PhysRevB.71.224429 (2005).

21 Bergeard, N. *et al.* Dynamic fluctuations and two-dimensional melting at the spin reorientation transition. *Phys. Rev. B* **86**, 094431, doi:10.1103/PhysRevB.86.094431 (2012).

22 Kronseder, M. *et al.* Real-time observation of domain fluctuations in a two-dimensional magnetic model system. *Nat. Commun.* **6**, 6832, doi:10.1038/ncomms7832 (2015).

23 Jenkins, A. *et al.* Single-spin sensing of domain-wall structure and dynamics in a thin-film skyrmion host. *Phys. Rev. Mater.* **3**, 083801, doi:10.1103/PhysRevMaterials.3.083801 (2019).
